# Weight changes according to treatment in a diverse cohort of breast cancer patients

**DOI:** 10.1186/s12885-021-08740-5

**Published:** 2021-09-08

**Authors:** Jami Fukui, Kami White, Timothy B. Frankland, Caryn Oshiro, Lynne Wilkens

**Affiliations:** 1 University of Hawaii Cancer Center, 701 Ilalo Street, Honolulu, HI 96813 USA; 2 Kaiser Permanente, Center for Integrated Health Care Research, 501 Alakawa St. Suite 201, Honolulu, HI 96817 USA

**Keywords:** Breast cancer, Weight changes, Sarcopenia, Geriatric oncology, Race/ethnicity

## Abstract

**Background:**

Weight changes are common among breast cancer patients. The majority of studies to date have focused on weight gain after a breast cancer diagnosis and its implications on health in survivors. Fewer studies have examined weight loss and its related characteristics. Weight changes have been reported to be influenced by several factors such as age, treatment, stage and pre-diagnostic weight. We evaluated weight changes during key treatment time points in early stage breast cancer patients.

**Methods:**

We characterized 389 female patients diagnosed in Hawaii with early stage breast cancer from 2003 to 2017 in the Multiethnic Cohort (MEC) linked with Kaiser Permanente Hawaii electronic medical record data. We evaluated weight changes from surgery to 4 years post-diagnosis with six time points along a patient’s treatment trajectory (chemotherapy, radiation, endocrine, or surgery alone) and annually thereafter, adjusting for age, race/ethnicity and initial body mass index (BMI).

**Results:**

We found key time points of significant weight change for breast cancer patients according to their adjuvant treatment. In patients who had surgery alone (S), surgery-radiation (SR), or surgery-endocrine therapy (SE), the majority of patients had stable weight, although this consistently decreased over time. However, the percentages of patients with weight loss and weight gain during this time steadily increased up to 4 years after initial surgery. Weight loss was more common than weight gain by about 2 fold in these treatment groups. For patients with surgery-chemotherapy (SC), there was significant weight loss seen within the first 3 months after surgery, during the time when patients receive chemotherapy. And this weight loss persisted until year 4. Weight gain was less commonly seen in this treatment group.

**Conclusions:**

We identified key time points during breast cancer treatment that may provide a therapeutic window to positively influence outcomes. Tailored weight management interventions should be utilized to promote overall health and long term survivorship.

## Background

Obesity at diagnosis of breast cancer is an established negative prognostic factor and several studies report an inverse relationship between weight gain post diagnosis and disease free survival [1–3]. Weight gain is more common in younger women [4, 5] and women receiving adjuvant therapy [6, 7]. Schvartsman et al. found age differences related to weight changes during adjuvant chemotherapy, where women over 50 years old were more likely to lose weight during adjuvant chemotherapy, whereas women under 30 years old gained weight [8]. In one study, Asian women had the lowest risk of weight gain post breast cancer diagnosis [5].

The evaluation of body weight and breast cancer recurrence has been predominantly based on risk associated with excess body weight rather than on the risk associated with weight loss. Sarcopenia, a loss of muscle mass or strength, can naturally occur with aging and is further exacerbated in older adults with cancer. The prevalence of sarcopenia is between 12 to 57% in older cancer patients [9]. Cancer treatments such as surgery, chemotherapy, and radiation may further contribute to muscle loss [10]. Sarcopenia is as common and important a risk factor as obesity for cancer outcomes, including survival, chemotherapy toxicity and surgical outcomes [11]. In a recent meta-analysis, sarcopenia was found to be a risk factor for mortality among early stage female breast cancer patients [12]. However a study by Del Fabbro et al. examined sarcopenia in women with early stage breast cancer and found it to be associated with improved outcomes [13].

The majority of prior studies evaluated weight change for women at a median age over 55 in early stage breast cancer 6 months or more post diagnosis. Vagenas et al. found that weight gain can continue to increase with time post-diagnosis, where the percentage overweight or obese was more than half (57%) of patients at 6 months post-surgery, and increased to 68% by 72 months post-surgery [6]. In a sample of 625 women with early breast cancer, 31% lost about 5 lbs., 34% had a stable weight, and 35% gained about 5 lbs. at 2 years post-primary treatment (surgical, radiation or chemotherapy) [4]. In women diagnosed with stage I – III invasive breast cancer with chemotherapy and/or radiation treatment, 19% experienced weight loss, 62% remained stable, and 19% gained weight at 18 months post diagnosis [14]. In a study of Asian women with stage 0 – IV breast cancer, 27% lost weight, 12% remained stable, and 61% gained weight from diagnosis to 18 months post diagnosis [15].

Inconsistency exists between studies that examine weight changes after a breast cancer diagnosis due to variability in population, cancer stage, cancer related treatments, definition of weight change and the time point at which weight change is assessed, making comparisons difficult. Moreover, very few studies examining weight loss after diagnosis and treatment are described in older age groups, over multiple time points, and/or in a multiethnic population, including Native Hawaiians/Pacific Islanders, an understudied group and at high risk for breast cancer incidence and mortality [16].

Our study evaluates changes in weight from surgery to 4 years post-diagnosis, across six time points along a patient’s treatment trajectory; chemotherapy, radiation, endocrine treatment, or surgery alone, adjusting for age, race/ethnicity, and BMI at surgery.

## Methods

### Study population

The Multiethnic Cohort (MEC) was designed to study lifestyle and genetic factors in relation to cancer and other chronic diseases [17]. Between 1993 and 1996 more than 215,000 men and women aged 45–75 years, mainly African American, Native Hawaiian, Japanese American, Latino, or White, and living in Hawaii or California were enrolled in the MEC by completing a 26-page mailed questionnaire on diet, medical history, and lifestyle. Cancer cases are identified through annual linkage to the statewide SEER registries, the Hawaii Tumor Registry (HTR) and the California Cancer Registry. To improve information on treatment and other aspects of cancer care, a linkage was performed for Hawaii (HI) MEC participants and the Kaiser Permanente Hawaii (KPHI) electronic medical records (EMR) for years 2000–2017. The subset of MEC female breast cancer cases in KPHI with EMR anthropometry data and undergoing surgery is the focus of this manuscript.

**Fig. 1:**
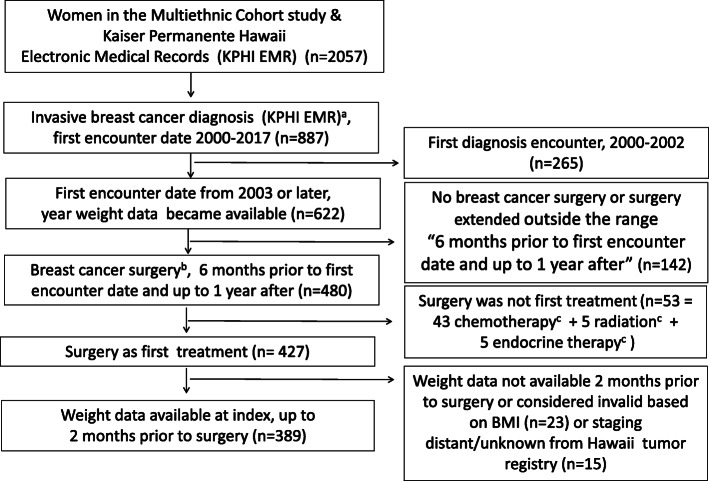
Flowchart of Hawaii cases from the Multiethnic Cohort (MEC)-Kaiser linked dataset. Study sample selection. ^a^Defined using International Classification of Diseases (ICD) 9th/10th revision codes C50, 174, Z85.3, V10.3. ^b^Defined using ICD-9/10, Current Procedural Terminology (CPT), and local Kaiser Permanente Hawaii (KPHI) codes. ^c^Defined using ICD 9/10, CPT, Healthcare Common Procedure Codes, Revenue codes, and National Drug Codes

Among 2057 MEC female cancer cases linked to KPHI, 887 were diagnosed with invasive breast cancer and had a first encounter/visit date for that diagnosis (with ICD 9/10 codes for invasive breast cancer) occurring from 2000 to 2017 (see Fig. 1). Patients with first encounter dates before 2003 were excluded (*n* = 265) as anthropometric data only became available in the KPHI EMR in that year.

### Treatment

Only patients who had surgery due to breast cancer, primarily lumpectomy or mastectomy during the period from 6 months prior to diagnosis encounter date, to 1 year after, were included (*n* = 480). All treatments received within ±1 year of surgery were identified in the EMR. The order of treatments (surgery, chemotherapy, radiation, or endocrine therapy) was determined, and only patients who had surgery as their first treatment were selected for analysis (*n* = 427). Surgery date was assigned as the index date. To evaluate the effect of different treatments on weight outcomes we classified treatment as surgery alone (S), surgery followed by radiation (SR) with and without additional endocrine therapy, surgery followed by chemotherapy (SC), and surgery followed by endocrine therapy (SE).

### Weight measures

Body mass index (BMI) was first calculated at each patient’s encounters using the mean height of all encounters (average number of encounters for the 427 patients with surgery as their first treatment = 21, mean standard deviation of height values = 0.7 in.), and used to identify implausible weight measures; in particular weights were excluded if the corresponding BMI value was outside the range of 13–60 kg/m^2^. Time from the date of surgery (index date) to the date of measured weight was computed and assigned to the following 7 time points and associated time periods: 0 = 2 months prior to and up to the index date, 1 = 0.1 to 2.9 months after index date, 2 = 3 to 5.9 months after, 3 = 6 to 11.9 months after, 4 = 12 to 23.9 months after, 5 = 24 to 35.9 months after, and 6 = 36 to 47.9 months after. Weight measures were averaged within each time period for each patient, and patients who were missing a weight measure at the index time point 0 were excluded. The difference in weight from the index time period was calculated for each subsequent time point and categorized as follows: Lost weight (at least 5 lbs), Stable (lost/gained within 5 lbs), and Gained weight (5 lbs. or more). The final study sample included 389 patients.

### Race/ethnicity

Based on KPHI EMR data, patients were classified into four race/ethnic groups for this analysis: Native Hawaiian/Pacific Islander (NHPI), Asian, White, or Other/Unknown. Patients who identified as part American Indian/Alaskan Native or Black or who were unknown (*n* = 10) were assigned “Other/Unknown”. The remaining patients were assigned, in priority, as NHPI if they identified as any part Native Hawaiian/Pacific Islander, “Asian” if identified as any part Asian, and “White” if identified as White. We did not consider Hispanic origin in the classification given the low prevalence of patients with Hispanic ethnicity in Hawaii.

### Other measures

Other covariates of interest from the MEC data included sex, education, neighborhood socioeconomic status (nSES) [18, 19], smoking status, dietary intake, physical activity, and reproductive history. The KPHI EMR data was supplemented with HTR data for staging, grade, lymph node involvement, and ER/PR status for the breast cancer diagnosis, as the SEER variables are abstracted and defined uniformly. This study was reviewed and approved by the University of Hawaii and Kaiser Permanente Hawaii Institutional Review Boards for human subjects research.

### Statistical analysis

Mixed generalized odds logistic regression was used to evaluate patient weight changes from surgery (outcomes: lost weight, kept stable weight, gained weight) to six time points. A random intercept term for each patient and fixed effects for time (6 levels: < 3, 4–5, 6–11, 12–23, 24–35, 36–47 months), age at surgery (3 levels: < 65, 65–74, 75+ years), race (4 levels: Native Hawaiian/Pacific Islander, Asian, White, Other/unknown), and BMI at surgery (4 levels: < 25, 25–29.9, 30–34.5, 35+ kg/m^2^), were included in the model. The predicted probability for each outcome for each patient was obtained and averaged across all patients for each time period from surgery. To identify possible differences in weight changes by treatment, a similar model was run, including fixed effects for treatment type (4 levels: surgery alone, surgery-chemotherapy, surgery-radiation, surgery-endocrine therapy) and cross-product terms for the interaction of time and treatment type. Fixed effects were assessed using the F test, and two-sided *p*-values < 0.10 for the interaction term was considered suggestive. All analyses were repeated including only patients with complete data at all time points (overall: *n* = 248 patients; by treatment type: surgery alone, *n* = 33, surgery-chemotherapy, *n* = 43, surgery-radiation, *n* = 94, surgery-endocrine therapy, *n* = 78). Because results from both samples were similar, results from the full sample are reported here. All analyses were performed using SAS version 9.4.

## Results

### Sample characteristics

As shown in Table 1, the female breast cancer patients were evenly distributed (~ 30%) among Native Hawaiians/Pacific Islanders (NHPI), Asians, and Whites, which is roughly consistent with the racial/ethnic diversity of Hawaii. Mean age at surgery of the overall cohort was 71.1 years with a range of 54–94 years. NHPI and White patients were younger, whereas Asian patients were older. Mean body mass index (BMI) at surgery was 28.3 kg/m^2^ for the entire cohort; NHPI patients had the highest mean BMI and Asian patients the lowest (31.7 and 26.2 kg/m^2^, respectively).

**Table 1:** Patient characteristics at Breast Cancer surgery

Patient Characteristics at Surgery, n (%)	All	NHPI	Asian	White	Other/Unknown
N	389	118	132	129	10
Age, years, mean (SD)	71.1 (7.8)	69.9 (7.9)	73.2 (7.7)	70.1 (8.8)	71.7 (8.8)
< 65	86 (22.1)	31 (26.3)	22 (16.7)	31 (24.0)	2 (20.0)
65–74	177 (45.5)	55 (46.6)	53 (40.2)	66 (51.2)	3 (30.0)
75+	126 (32.4)	32 (27.1)	57 (43.1)	32 (24.8)	5 (50.0)
Height, inches, mean (SD)	62.6 (2.8)	63.6 (2.6)	60.6 (2.3)	63.8 (2.3)	63.1 (4.2)
Weight, lbs., mean (SD)	158.4 (41.9)	182.5 (45.3)	136.7 (30.1)	157.8 (35.1)	167.0 (35.1)
BMI, kg/m^2^, mean (SD)	28.3 (6.7)	31.7 (7.1)	26.2 (5.3)	27.3 (6.1)	29.6 (10.5)
< 25	129 (33.2)	18 (15.3)	61 (46.2)	47 (36.4)	3 (30.0)
25–29.9	126 (32.3)	35 (29.7)	45 (34.1)	43 (33.3)	3 (30.0)
30–34.9	75 (19.3)	28 (23.6)	18 (13.6)	26 (20.2)	3 (30.0)
35+	59 (15.2)	37 (31.4)	8 (6.1)	13 (10.1)	1 (10.0)

Table 2 presents characteristics of the patients’ breast cancer tumors. All 389 patients had early stage breast cancer, defined as invasive breast cancer in KPHI EMR. The average tumor size was 18.7 mm, with moderately differentiated grade and lymph node negative disease. The majority of patients had estrogen receptor (ER) and progesterone receptor (PR) positive disease (84.1 and 74.0%, respectively) with 40.4% of cases having unknown HER2 status. NHPI patients were more likely to have regional stage, when compared to Asian and White patients (28% vs 20 and 19%, respectively), and to have lymph node positive disease (26% vs 17 and 16%, respectively).

**Table 2:** Tumor characteristics

Tumor Characteristics, N (%)	All	NHPI	Asian	White	Other/Unknown
Stage					
In situ	61 (15.7)	15 (12.7)	25 (18.9)	18 (14)	3 (30)
Localized	241 (62)	70 (59.3)	80 (60.6)	87 (67.4)	4 (40)
Regional	87 (22.4)	33 (28)	27 (20.5)	24 (18.6)	3 (30)
Tumor size, mm, mean (SD)	18.7 (14.5)	19.1 (16.8)	18.9 (15.4)	17.7 (11.3)	24.7 (11.9)
Grade					
Well differentiated	78 (20.1)	25 (21.2)	25 (18.9)	28 (21.7)	0
Moderately differentiated	183 (47)	58 (49.2)	67 (50.8)	55 (42.6)	3 (30)
Poorly differentiated	90 (23.1)	25 (21.2)	28 (21.2)	34 (26.4)	3 (30)
Undifferentiated	12 (3.1)	5 (4.2)	3 (2.3)	4 (3.1)	0
Unknown	26 (6.7)	5 (4.2)	9 (6.8)	8 (6.2)	4 (40)
Lymph node					
Positive	77 (19.8)	31 (26.3)	22 (16.7)	21 (16.3)	3 (30)
Negative	309 (79.4)	87 (73.7)	107 (81.1)	108 (83.7)	7 (70)
Unknown	3 (0.8)	0	3 (2.3)	0	0
Estrogen receptor status					
Positive	327 (84)	102 (86.4)	108 (81.8)	109 (84.5)	8 (80)
Negative	52 (13.4)	13 (11)	19 (14.4)	18 (14)	2 (20)
Borderline	1 (0.3)	1 (0.9)	0 (0)	0 (0)	0 (0)
Unknown	9 (2.3)	2 (1.7)	5 (3.8)	2 (1.6)	0 (0)
Progesterone receptor status					
Positive	288 (74)	93 (78.8)	94 (71.1)	94 (72.9)	7 (70)
Negative	89 (22.9)	23 (19.5)	31 (23.5)	32 (24.8)	3 (30)
Borderline	2 (0.5)	0 (0)	1 (0.8)	1 (0.8)	0 (0)
Unknown	10 (2.6)	2 (1.7)	6 (4.6)	2 (1.6)	0 (0)
Her2 status					
Positive	20 (5.1)	3 (2.5)	10 (7.6)	6 (4.7)	1 (10)
Negative	210 (54)	66 (55.9)	62 (47)	77 (59.7)	5 (50)
Borderline	2 (0.5)	0	0	2 (1.6)	0 (0)
Unknown	157 (40.4)	49 (41.5)	60 (45.5)	44 (34.1)	4 (40)

Table 3 shows the patients’ treatment regimen following surgery. The majority of patients had either surgery followed by radiation or surgery followed by endocrine therapy, 35 and 31%, respectively. The primary treatment differed by race/ethnic group, with the most common being surgery-endocrine for NHPIs (36%) and surgery-radiation for Asians (37.9%) and Whites (38.0%). Surgery only and surgery followed by chemotherapy were less common primary treatments for the whole cohort, at 18 and 15% respectively.

**Table 3:** Treatment regimen

Treatment order, N (%)	All	NHPI	Asian	White	Other/Unknown
Surgery Only (S)	71 (18.3)	19 (16.1)	21 (15.9)	27 (20.9)	4 (40)
Surgery-Chemotherapy (SC)	59 (15.2)	19 (16.1)	18 (13.6)	20 (15.5)	2 (20)
Surgery-Radiation (SR)	137 (35.2)	38 (32.2)	50 (37.9)	49 (38)	0
Surgery-Endocrine therapy (SE)	122 (31.4)	42 (35.6)	43 (32.6)	22 (25.6)	4 (40)

**Fig. 2:**
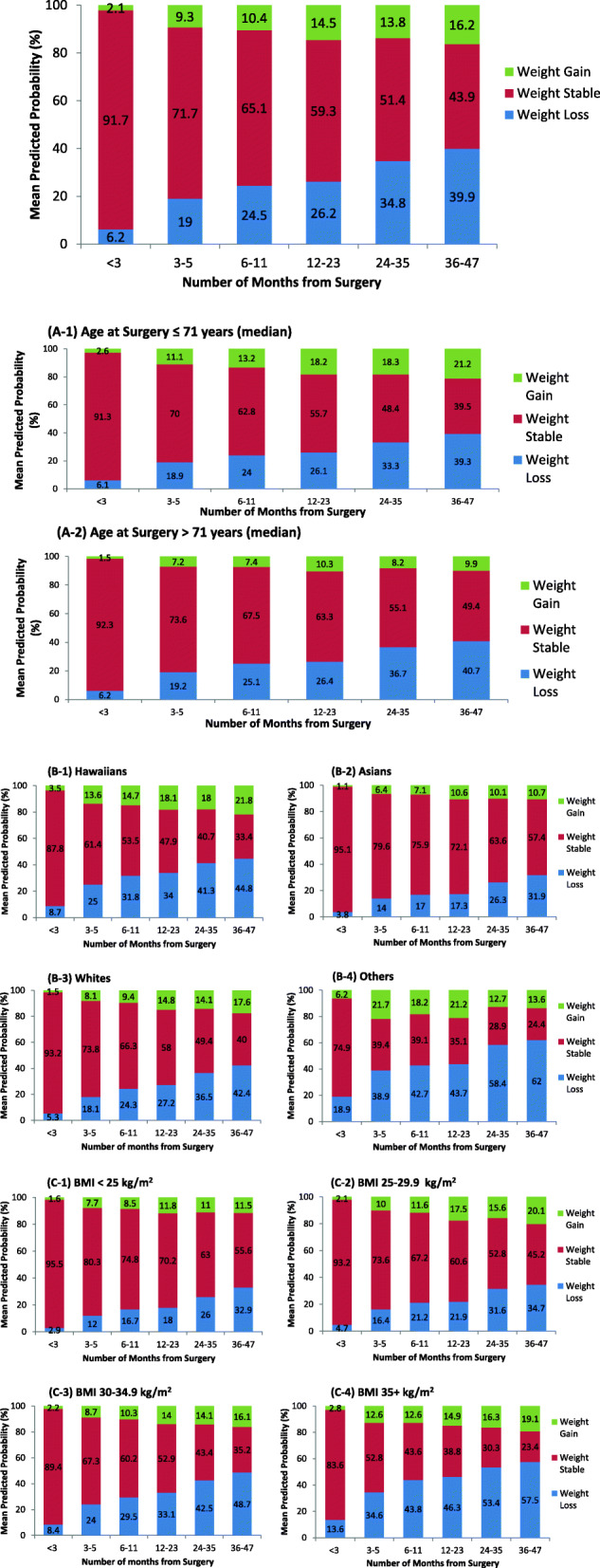
Weight changes from surgery based on generalized odds logistic model with outcomes loss/stable/gained weight, random intercept term for each patient, and fixed effects for time (6 groups, *p* < 0.0001), age at surgery (< 65, 65–74, 75+, *p* = 0.0009), race (Hawaiian, Asian, White, Other/unknown, *p* = 0.041), BMI at surgery (< 25, 25–29.9, 30–34.9, 35+, *p* = 0.0009). **A** (1, 2). Weight changes from surgery based on generalized odds logistic model described in Fig. 2. Number of months from surgery (< 3 months, 3–5, 6–11, 12–23, 24–35, 36–47 months): Fig. 2A-1, Age ≤ 71 years; Fig. 2A-2, Age > 71 years. **B** (1–4). Weight changes from surgery based on generalized odds logistic model described in Fig. 2. Number of months from surgery (< 3 months, 3–5, 6–11, 12–23, 24–35, 36–47 months): Fig. 2B-1, Hawaiians; Fig. 2B-2 Asians; Fig. 2B-3, Whites; Fig. 2B-4, Others. **C** (1–4). Weight changes from surgery based on generalized odds logistic model described in Fig. 2. Number of months from surgery (< 3 months, 3–5, 6–11, 12–23, 24–35, 36–47 months): Fig. 2C-1, BMI < 25 kg/m^2^; Fig. 2C-2, BMI 25–29.9 kg/m^2^; Fig. 2C-3, BMI 30–34.9 kg/m^2^; Fig. 2C-4, BMI 35+ kg/m^2^

Weight changes among all 389 patients across six time points, after adjustment for race/ethnicity, age and BMI at surgery are displayed in Fig. 2. The mean predicted probability for patients maintaining their weight < 3 months from surgery was over 90%; however, this percent steadily decreased to 44% at 36–47 months, with weight loss and weight gain at 40 and 16%, respectively. We compared this to the normal MEC cohort population with similar demographics as the MEC-KPHI breast cancer patients according to ethnicity (Hawaiian, Japanese = Asian, White), age range, BMI range, and first year of diagnosis (2003) to match the year of MEC data collection. We saw significant differences in weight change between the two groups. Based on polytomous logistic model with outcomes loss/stable/gained weight, fixed effects for time (5 groups, *p* < 0.0001), age (< 65, 65–74, 75+, *p* < 0.0001), race (Hawaiian, Japanese, White, *p* < 0.0001), BMI (< 25, 25–29.9, 30–34.9, 35+, *p* < 0.0001), during the time period, 34% of MEC participants lost weight compared to 40% of MEC-KPHI patients during the same time period.

Additionally, weight changes across time by age at surgery (groups divided at the median age 71 years), race/ethnicity, and BMI groups are shown In Fig. 2a, b, c, respectively.

Age at surgery (≤ 71 vs > 71 years, see Fig. 2a): The mean predicted probability of patients who maintained their weight steadily decreased across time for both age groups, though lower percentages were observed in patients age ≤ 71 years at surgery compared to patients age > 71 years. A higher percentage of patients who were age ≤ 71 years at surgery gained weight across time.

Race/Ethnicity (NHPI, Asians, Whites, Others, see Fig. 2b): The percentage of patients who had stable weight decreased across time across all race/ethnic groups. NHPIs and Whites, and to a lesser extent Asians, showed similar profiles, however NHPI had a higher percentage of patients who gained weight across time compared to Whites and Asians.

BMI at surgery (< 25, 25–29.9, 30–34.9, 35+ kg/m^2^, see Fig. 2c): Across all BMI groups, the percentage of patients who maintained their weight decreased across time. More patients with BMI 30+ kg/m^2^ (at surgery) loss weight across time compared to patients with BMI < 30 kg/m^2^.

**Fig. 3:**
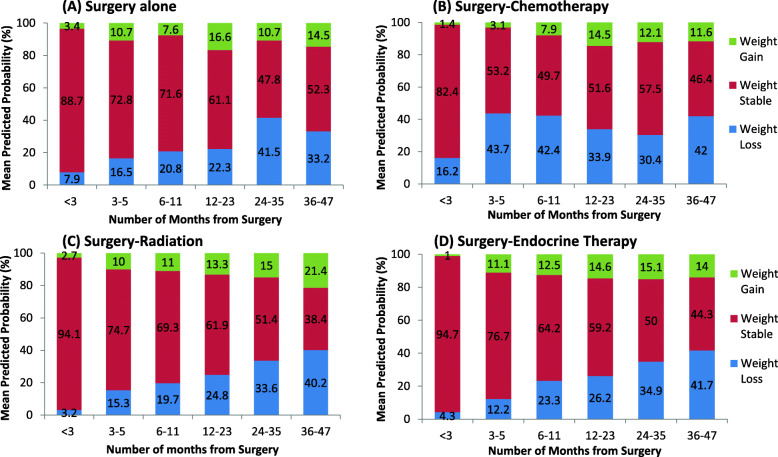
**A**-**D** Weight changes from surgery based on logistic model with outcomes loss/stable/gained weight, random intercept term for each patient, fixed effects for time (6 groups, *p* < 0.0001), age at surgery (< 65, 65–74, 75+, *p* = 0.0004), race (Hawaiian, Asian, White, Other/unknown, *p* = 0.055), BMI at surgery (< 25, 25–29.9, 30–34.9, 35+, *p* = 0.0009), treatment (Surgery alone (S), Surgery-Chemotherapy (SC), Surgery-Radiation (SR), Surgery-Endocrine therapy (SE), *p* = 0.073), interaction of time and treatment (*p* = 0.074).; Fig. 3B-SC; Fig. 3C-SR; Fig. 3D-SE

Figure 3 shows the mean predicted probability for the three outcomes (weight loss, stable weight, weight gain) across time by treatment group: Surgery alone (S), surgery followed by chemotherapy (SC), surgery followed by radiation (SR), and surgery followed by endocrine therapy (SE). Differences between treatment groups and time were suggestive (*p* = 0.07 for interaction term based on F with 30 degrees of freedom).

(S) Surgery alone (see Fig. 3a): The percentage of patients who maintained a stable weight from surgery generally decreased over time (89% at < 3 months to 52% at 36–47 months); patients with stable weight comprise the majority at each time point. Weight loss continued throughout the evaluated time frame and peaked 24–35 months after surgery at 41%. The percentage of patients with weight gain was generally consistent throughout this timeframe. We also ran the model according to surgery type (lumpectomy, mastectomy, other), with age, race, and BMI, and found that surgery type was not significant (*p* = 0.89), and the weight change profiles were similar.

(SC) Surgery-Chemotherapy (see Fig. 3b): From 3 months on, the percentage of patients who maintained weight after surgery was generally constant at roughly 50%. Within 3 months after surgery, a time when patients routinely receive chemotherapy, 16% of patients had significant weight loss, and over 30% of patients had weight loss at each subsequent time point. Weight gain continued to steadily increase during this timeframe and peaked 12–23 months after surgery at 14%. Weight gain was less commonly seen than weight loss throughout the post-surgery period.

(SR) Surgery-Radiation (see Fig. 3c): The percentage of patients who maintained a stable weight from surgery consistently decreased over time (94% at < 3 months to 38% at 36–47 months), and the percentage of patients who loss or gained weight steadily increased after initial surgery. Weight loss was about 2-fold more common than weight gain at each time point.

(SE) Surgery-Endocrine (see Fig. 3d): The pattern of weight change in this patient group was very similar to the pattern in the surgery-radiation patient group, where the percentage of patients with stable weight decreased over time.

Weight changes from surgery were similar between the treatment groups of S, SR and SE across time (*p* = 0.89 based on a F test with 20 degrees of freedom). A similar model that included all 389 patients and a modified treatment term, collapsing S, SR, SE into one group (vs SC), resulted in a significant interaction term between time and treatment (*p* = 0.0008 based on a F test with 10 degrees of freedom). Patients who underwent SC treatment experienced different weight changes compared to S, SR, and SE groups; they had the highest percent weight loss, particularly within the first 3 months after surgery, while they received chemotherapy. This percentage with weight loss plateaued at 3–5 months and continued for the remainder of the timeframe, up to 4 years after surgery.

## Discussion

We evaluated 389 early stage MEC/KPHI breast cancer patients’ weight changes for 4 years after surgery according to treatment—chemotherapy, radiation, endocrine therapy or surgery alone—adjusted for age, race/ethnicity and initial BMI. Our overall population is older than the median age of breast cancer diagnosis, due to the MEC age criteria of 45–75 in the mid-90’s and is racially/ethnically diverse. Consistent with known racial/ethnic disparities, this group of NHPI patients had proportionately more regional stage and lymph node positive disease, compared to Asian and White patients. When adjusted for age at diagnosis, race/ethnicity and initial BMI, we saw treatment related weight change differences. Overall, all treatment groups (S, SC, SR, SE) showed that a majority of patients had stable weight. However over time, weight loss compared to weight gain continued to increase and was comparatively over 2-fold higher.

We suspect the ongoing weight loss in all treatment groups to be at least in part due to sarcopenia. This can naturally occur with aging and is further exacerbated in older adults with cancer. There are recent efforts to focus on body composition evaluation using CT technology and correlate metrics of skeletal muscle mass, visceral adipose tissue, subcutaneous adipose tissue and others with breast cancer outcomes [20, 21]. For breast cancer patients receiving chemotherapy, sarcopenia has been found a predictor of adverse events [22]. Also, in a recent meta-analysis, sarcopenia was associated with not only severe chemotherapy toxicity as well as shorter overall survival and time to tumor progression. All together this suggests that in clinical practice, body composition assessment could be valuable as a prognostic parameter in breast cancer. Despite being a risk factor for mortality among early stage female breast cancer patients, there are no guidelines for ongoing or initial evaluation of patients for sarcopenia. Clinical trial interventions evaluating the impact of diet and physical activity on weight gain after a breast cancer diagnosis have been underway [23–25]. More recent interventions are utilizing body composition analysis to evaluate improvement in muscle mass and function in breast cancer survivors. We have a tailored exercise program for breast cancer patients, irrespective of their current treatment, and have utilized body composition metrics to evaluate changes and correlate them with disease outcomes (NCT04013568).

The majority of prior studies evaluated weight change in early stage breast cancer at least 6 months post diagnosis. In our study we utilized all available weights and assigned them to established treatment time points. Despite completing a majority of adjuvant treatments (chemotherapy and radiation) by 12 months after surgery, continued weight loss was observed in all treatment groups. The differences between weight loss and weight gain increased and worsened from 12 months to 48 months for the treatments of surgery (S), surgery-radiation (SR) and surgery-endocrine (SE).

The surgery-chemotherapy (SC) treatment group showed significant differences between weight loss and weight gain initially, beginning at month 3 that persisted to 48 months, indicating a significant difference from other treatment groups.

This initial steep increase in percent of patients with weight loss for those who received SC, reveals a potentially important therapeutic window for intervention. In practice, patients who are undergoing chemotherapy are encouraged to stay physically active as tolerated during their treatment; however there are no established guidelines that are commonly recommended. Often due to chemotherapy toxicities including fatigue, nausea/vomiting, and decreased appetite, patients struggle to be physically active and this can further contribute to deconditioning. Our group is assessing a tailored exercise program for breast cancer patients undergoing various treatments (S, SR, SE, SC). Other studies have evaluated physical activity regimens during chemotherapy and found improvements in cardiorespiratory fitness [26] chemotherapy related fatigue [27] and less nausea and pain [28]. Our data suggest the initial 3 months post initial surgical intervention to be the most dynamic and potentially important period for addressing weight loss changes in patients with early stage breast cancer undergoing chemotherapy. Efforts should be made to utilize local physical activity programs and/or accrue to available clinical trials that intervene during this timeframe.

### Strengths and Limitations

Our study utilizes a substantial amount of data from three independent and unique sources: HI MEC, KPHI and HTR. Several coding sources (ICD-9/10, CPT, HCPC, local Kaiser codes) in the KPHI EMR were used to identify breast cancer treatment (surgery, chemotherapy, radiation, and endocrine therapy). Through a comprehensive review of all procedure and prescription codes in patients’ EMR, 6 months prior to their first encounter/diagnosis date and up to 1 year after, as well as manual chart review for some, we appropriately classified the different types of breast cancer treatment, despite each type being coded in a variety of ways. With data available at each patient encounter, we also had access to an abundance of data, including enrollment, demographics, diagnosis, and detailed treatment. Diagnosis data from KPHI EMR and HTR were generally consistent as well. In addition, our diverse and older population fills a gap currently lacking in the literature.

There are some limitations however, our study did not obtain information regarding whether there was intentionality for weight loss. Because these data were adjusted for initial BMI and still weight loss was more prevalent than weight gain, we conclude that this trend represents all patients—overweight, underweight and normal weight at initial diagnosis. Although we have some information on physical activity and dietary habits from the MEC participants at date of cohort entry and 10 years afterward, we do not have information on specific behavioral changes occurring during treatment. Additionally our observation period post treatment is up to 4 years, which may not capture a long enough time frame to conclude effects on breast cancer survivors. Also, no recurrence data was available to evaluate if weight changes had any effect on disease free survival in this cohort.

## Conclusions

Our study found treatment related differences in weight changes irrespective of age, race/ethnicity and initial BMI. Although the majority of patients had stable weight after initial surgery, weight loss increased irrespective of treatment, S, SC, SR, SE. Given the adverse consequences of weight gain and weight loss after breast cancer diagnosis, particularly for those undergoing chemotherapy, identifying interventional strategies during treatment may positively influence outcomes. Targeted weight management interventions should be utilized during and after treatment to promote overall health and long term survivorship.

## Data Availability

Data are available from the Multiethnic Cohort study (http://www.uhcancercenter.org/mec-researchers/mec-data-sharing) for researchers who meet the criteria for access to confidential data. For details and to request application template please contact Gail Ichida, gichida@cc.hawaii.edu.

## Abbreviations

EMR: Electronic Medical Record
HI-MEC: Hawaii Multiethnic Cohort
HTR: Hawaii Tumor Registry
KPHI: Kaiser Permanente Hawaii
NHPI: Native Hawaiian/Pacific Islanders
